# Supplementary material for: Antimicrobial Stewardship for Outpatients with Chronic Bone and Joint Infections in the Orthopaedic Clinic of an Academic Tertiary Hospital, South Africa
Source: Antibiotics (Basel). 2023 Jul 1;12(7):1142. doi: 10.3390/antibiotics12071142 (PMC10376089; doi:10.3390/antibiotics12071142)
Supplement: Supplementary file 1 [file antibiotics-12-01142-s001.zip › antibiotics-2420050-supplementary.pdf]

antibiotics

MDPI

Supplementary Material

Table S1. Adherence to Refills and Medication scale (ARMS) questionnaire.

Questions	None of the time	Some of the time	Most of the time	All of the time
How often do you forget to take your medicine?				
How often do you decide not to take your medicine?				
How often do you forget to get prescriptions filled?				
How often do you run out of medicine?				
How often do you skip a dose of your medicine before you go to the doctor?				
How often do you miss taking your medicine when you feel better?				
How often do you miss taking your medicine when you feel sick?				
How often do you miss taking your medicine when you are not paying attention to your regimen?				
How often do you change the dose of your medicines to suit your needs (like when you take more or less pill than you're supposed to)?				
How often do you forget to take your medicine when you are supposed to take it more than once a day?				
How often do you put off refilling your medicines because of cost?				
How often do you plan ahead and refill your medicines before they run out?				

Table S2. Knowledge of the condition and medication checklist.

**Questions**	**Possible answers (Tick "√")**	**Other**	**"Don't Know"**
**Knowledge of condition**			
What is the name of your condition/what is the diagnosis of your condition?	Bone infection<br>Osteomyelitis<br>Septic Arthritis		
What are the common signs and symptoms of your condition?	Tenderness of the affected area<br>Pain<br>Swelling<br>Fever<br>Chills<br>Decreased motion<br>Discomfort		
What caused your condition?	Infection<br>Trauma<br>Genetics<br>Age		
**Knowledge of medication**			
What is the purpose of your medicine treatment?	Eradicate infection<br>Alleviate pain/<br>Antibiotic - it eradicates the infection by killing the bacteria<br>Analgesic - Pain reliever and also reduces fever		
How many doses do you take?/ How many tablets do you take?	1<br>2<br>3<br>4		
How many times a day do you take your medication?	3 times a day<br>4 times a day		
Which route of administration do you use to administer your medications?	Oral		
What are the common side effects you can experience with your medication?	Antibiotics:<br>Gastrointestinal disturbances(GI) (stomach cramps, nausea, vomiting, diarrhoea), hypersensitivity reactions<br>Analgesics: GI disturbances		
For how long is your treatment going to be?	1 week<br>1 month<br>6 months		

Table S3 Demographics and clinical data form.

<table>
<tr><td colspan="6">Demographic details:</td></tr>
<tr><td colspan="6">Date:</td></tr>
<tr><td colspan="6">File number:</td></tr>
<tr><td>Level of education</td><td>Primary school</td><td>High school</td><td>University</td><td>Other institutions</td><td>No formal education</td></tr>
<tr><td colspan="2">Age (years):</td><td colspan="2">Sex (M/F):</td><td colspan="2">Occupation:</td></tr>
<tr><td colspan="6">Other chronic conditions:</td></tr>
<tr><td colspan="6">Clinical data:</td></tr>
<tr><td colspan="2">Diagnosis</td><td colspan="4"></td></tr>
<tr><td colspan="2">Date of Diagnosis</td><td colspan="4"></td></tr>
<tr><td colspan="2">Microbiological test findings</td><td colspan="4"></td></tr>
<tr><td colspan="2"></td><td colspan="4"></td></tr>
<tr><td colspan="2" rowspan="2">Prescribed treatment</td><td colspan="2">Treatment according to guideline</td><td colspan="2" rowspan="2">Treatment dispensed</td></tr>
<tr><td>YES</td><td>NO</td></tr>
<tr><td colspan="2">1.</td><td></td><td></td><td colspan="2"></td></tr>
<tr><td colspan="2">2.</td><td></td><td></td><td colspan="2"></td></tr>
<tr><td colspan="2">3.</td><td></td><td></td><td colspan="2"></td></tr>
<tr><td colspan="2">4.</td><td></td><td></td><td colspan="2"></td></tr>
<tr><td colspan="2">Intervention needed (tick “√”)</td><td colspan="2">YES</td><td colspan="2">NO</td></tr>
<tr><td colspan="2">Drug change</td><td colspan="4"></td></tr>
<tr><td colspan="2">Correct dose</td><td colspan="4"></td></tr>
<tr><td colspan="2">Correct duration</td><td colspan="4"></td></tr>
<tr><td colspan="2">Other</td><td colspan="4"></td></tr>
<tr><td colspan="2">Intervention accepted</td><td colspan="2">YES</td><td colspan="2">NO</td></tr>
</table>

Table S4. Organisms cultured and the treatment prescribed and dispensed after interventions.

Study No.	Cultures	Prescribed Antibiotic/s	Intervention Necessary	Motivation for Intervention	Antibiotic Dispensed
1	Coagulase negative *Staphylococcus aureus*	Cloxacillin 2g po qid	Yes	Drug change	Flucloxacillin 2g po qid
2	*Staphylococcus aureus*	Rifampicin 150mg po dly	Yes	Drug addition, Correct dose, Correct frequency	Rifampicin 300mg po bd Ciprofloxacin 500mg po bd
3	No microbiology tests conducted	Rifampicin 300mg po dly	Yes	Correct dose, Correct frequency	Rifampicin 300mg po bd
		Cloxacillin 1g po qid	Yes	Drug change	Flucloxacillin 1g po qid
4	*Staphylococcus aureus*	Flucloxacillin 2g po qid	No		Flucloxacillin 1g po qid
		Rifampicin 600mg po dly	No		
5	Coagulase negative *Staphylococcus aureus*	Cloxacillin 500mg po tds	Yes	Drug change	Ciprofloxacin 500mg po bd
		Rifampicin 300mg po dly	Yes	Correct frequency	Rifampicin 300mg po bd
6	No growth observed	Flucloxacillin 1g po qid	No		Flucloxacillin 1g po qid
7	*Staphylococcus aureus*	Flucloxacillin 2g po qid	No		Flucloxacillin 2g po qid
8	No growth observed	Flucloxacillin 2g po qid	No		Flucloxacillin 2g po qid
9	Coagulase negative *Staphylococcus aureus*	Flucloxacillin 2g po qid	No		Flucloxacillin 2g po qid
		Rifampicin 600mg po dly	No		
10	*Staphylococcus aureus*	Flucloxacillin 2g po qid	No		Flucloxacillin 2g po qid
11	No growth observed	Rifampicin 300mg po bd	No		Rifampicin 300mg po bd
		Ciprofloxacin 500mg po bd	No		Ciprofloxacin 500mg po bd
12	Methicillin Resistant *Staphylococcus aureus*	Ciprofloxacin 500mg bd	Yes	Not covered by antibiotic*	Ciprofloxacin 500mg po bd
		Rifampicin 300mg po bd			Rifampicin 300mg po bd
13	*Staphylococcus aureus*	Ciprofloxacin 500mg bd	No		Ciprofloxacin 500mg po bd
		Rifampicin 300mg po bd	No		Rifampicin 300mg po bd
14	No microbiology tests conducted	Ciprofloxacin 500mg bd	No		Ciprofloxacin 500mg po bd
		Rifampicin 300mg po bd	No		Rifampicin 300mg po bd

15	No growth observed	Amoxicillin./clavulanic acid 1.2g po tds	No		Amoxicillin/clavulanic acid 1g po tds
16	Methicillin resistant *Staphylococcus aureus*	Ciprofloxacin 500mg bd	Yes	Not covered by antibiotic*	Ciprofloxacin 500mg po bd
		Rifampicin 300mg po bd	No		Rifampicin 300mg po bd
17	*Pseudomonas aeuriginosa* and *Morganella morganii subsp morganii*	Cloxacillin 1g po qid	Yes	Drug change	Ciprofloxacin 750mg bd
18	No microbiology tests conducted	Flucloxacillin 1g po qid	No		Flucloxacillin 1g po qid
19	No microbiology tests conducted	Ciprofloxacin 500mg bd	No		Ciprofloxacin 500mg bd
		Rifampicin 300mg po bd	No		Rifampicin 300mg po bd
20	No microbiology tests conducted	Cloxacillin 1g po qid	Yes	Drug change	Flucloxacillin 1g po qid
21	No microbiology tests conducted	Ciprofloxacin 500mg bd	No		Ciprofloxacin 500mg bd
		Rifampicin 300mg po bd	No		Rifampicin 300mg po bd
22	No growth observed	Cloxacillin 1g po qid	Yes	Drug change	Flucloxacillin 1g po qid
23	*Pseudomonas aeuriginosa*	Ciprofloxacin 500mg bd	No		Ciprofloxacin 500mg bd
		Rifampicin 300mg po bd	No		Rifampicin 300mg po bd
24	*Proteus mirabilis* and *Escherichia coli*	Cloxacillin 1g po qid	No	Drug change	Ciprofloxacin 750mg bd
25	*Staphylococcus aureus*	Amoxicillin./clavulanic acid 1.2g po bd	No		Amoxicillin/clavulanic acid 1g po bd
26	*Pseudomonas aeuriginosa*	Cloxacillin 1g po qid	Yes	Drug change	Ciprofloxacin 750mg bd
		Rifampicin 300mg po bd	No		Rifampicin 300mg po bd
27	*Staphylococcus aureus*	Cloxacillin 1g po qid	Yes	Drug change	Flucloxacillin 1g po qid
28	No microbiology tests conducted	Ciprofloxacin 500mg bd	No		Ciprofloxacin 500mg bd
		Rifampicin 300mg po bd	No		Rifampicin 300mg po bd
29	No microbiology tests conducted	Ciprofloxacin 500mg bd	No		
		Rifampicin 300mg po bd	No		Rifampicin 300mg po bd
30	*Staphylococcus aureus* and *Enterobacter cloacae complex*	Cloxacillin 1g po qid	Yes	Drug change	Flucloxacillin 1g po qid
31	*Enterobacter cloacae complex*	Ciprofloxacin 500mg bd	No		Ciprofloxacin 500mg bd
		Rifampicin 300mg po bd	No		Rifampicin 300mg po bd

32	No growth observed	Cloxacillin 1g po qid	Yes	Drug change	Flucloxacillin 1g po qid
		Rifampicin 300mg po dly	Yes	Correct dose	Rifampicin 300mg po bd
33	No growth observed	Ciprofloxacin 500mg bd	No		Ciprofloxacin 500mg bd
		Rifampicin 600mg po dly	No		Rifampicin 600mg po dly
34	*Staphylococcus aureus*	Cloxacillin 1g po qid	Yes	Drug change	Flucloxacillin 1g po qid
35	No microbiology tests conducted	Ciprofloxacin 500mg bd	No		Ciprofloxacin 500mg bd
		Rifampicin 300mg po bd	No		Rifampicin 300mg po bd
36	No microbiology tests conducted	Ciprofloxacin 500mg bd	No		Ciprofloxacin 500mg bd
		Rifampicin 300mg po bd	No		Rifampicin 300mg po bd
37	*Escherichia coli*	Cloxacillin 1g po qid	Yes	Drug change	Flucloxacillin 1g po qid
		Rifampicin 300mg po dly	Yes	Correct dose	Rifampicin 300mg po bd
38	No microbiology tests conducted	Cloxacillin 1g po qid	No	Drug change	Flucloxacillin 1g po qid
39	*Staphylococcus aureus*	Flucloxacillin 2g po qid	No		Flucloxacillin 2g po qid
		Rifampicin 600mg po dly	No		Rifampicin 600mg po dly
40	No microbiology tests conducted	Ciprofloxacin 500mg bd	No		Ciprofloxacin 500mg bd
		Rifampicin 300mg po bd	No		Rifampicin 300mg po bd
41	No microbiology tests conducted	Flucloxacillin 1g po qid	No		Flucloxacillin 1g po qid
		Rifampicin 300mg po bd	No		Rifampicin 300mg po bd
42	*Proteus mirabilis*	Ciprofloxacin 500mg bd	No		Ciprofloxacin 500mg bd
		Rifampicin 300mg po bd	No		Rifampicin 300mg po bd
43	*Staphylococcus aureus*	Flucloxacillin 2g po qid	No		Flucloxacillin 2g po qid
		Rifampicin 600mg po dly	No		Rifampicin 600mg po dly
44	*Pseudomonas aeuriginosa*	Flucloxacillin 1g po tds	Yes	Drug change	Ciprofloxacin 500mg bd
		Rifampicin 300mg po bd	No		Rifampicin 300mg po bd

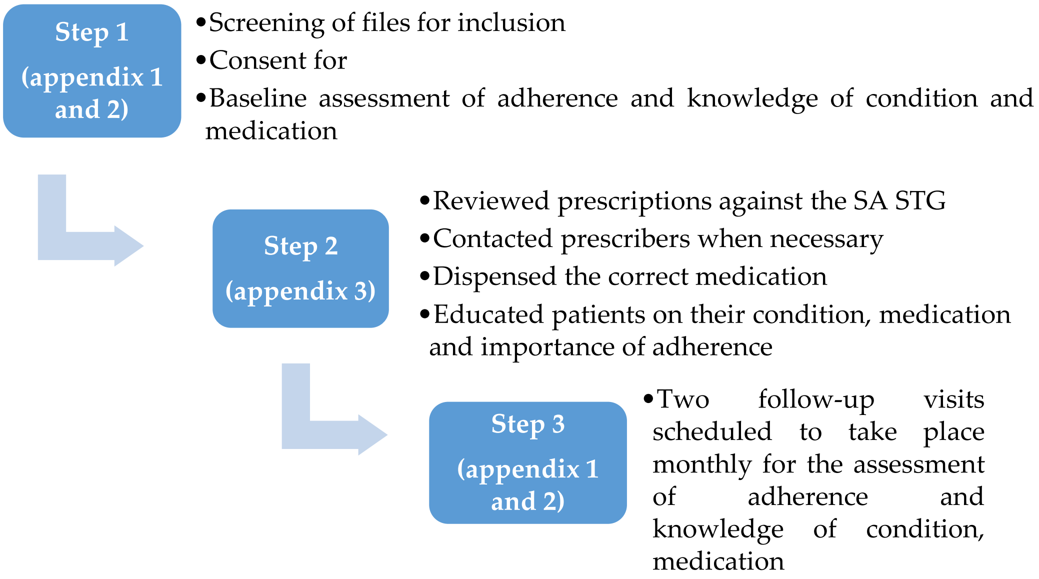

Figure S1. Flow diagram of data collection procedure.
